# Early Implementation of Collaborative Care in France: Facilitators and Barriers from the First Year of the SESAME Program

**DOI:** 10.5334/ijic.9845

**Published:** 2026-10-01

**Authors:** Irène Bogicevic, Milena Kostova, Pascal Clerc, Christine Passerieux, Coralie Gandre, Nadia Younes

**Affiliations:** 1 Laboratoire Paragraphe, Department of Psychology, UniversitéParis 8 Vincennes-Saint-Denis, France; 2 Maison de Santé Pluriprofessionnelle des Mureaux, France; 3 Department of General Practice, Université de Versailles Saint-Quentin-en-Yvelines, France; 4 Simone Veil Health Sciences Faculty, Université de Versailles Saint-Quentin-en-Yvelines, Versailles, France; 5 Department of Adult Psychiatry and Addictology, Centre Hospitalier de Versailles, Le Chesnay, France; 6 Quartet Santé, Paris, France; 7 Institute for Research and Information in Health Economics (IRDES), Paris, France; 8 Université Versailles Saint-Quentin, Université Paris-Saclay, INSERM U1018, DevPsy Team, Villejuif, France

**Keywords:** Collaborative Care Model, implementation research, primary care, facilitators and barriers, depression and anxiety care management, modèle de soins collaboratifs, recherche en implémentation, soins primaires, facilitateurs et obstacles, prise en charge de la dépression et de l’anxiété

## Abstract

**Background:**

The Collaborative Care Model (CoCM), which integrates mental health services into primary care through a team-based approach involving GPs, care managers (CM), and mental health consultants, is a proven way to meet these challenges. A first CoCM adaptation in French primary care settings called SESAME has been implemented since 2021.

**Methods:**

Semi-structured interviews were conducted with all the healthcare professionals involved in SESAME to examine the facilitators and barriers experienced during the first year. Data were analyzed using a qualitative approach combining manual thematic analysis and lexicometric analysis with ALCESTE software.

**Results:**

Four discourse classes emerged from the analysis. Key facilitators included prior collaborative experience, effective digital communication tools, and clear role understanding. Barriers such as technological challenges, role ambiguity, and referral framework misunderstandings were also identified. The structured inclusion process and rapid access to care were appreciated, but challenges persisted in referral processes and role boundary definitions. Financial coverage and a less stigmatizing approach were seen as benefits.

**Conclusion:**

This study provides insights into the early implementation dynamics of CoCM in a novel context, underscoring the need for clear protocols, ongoing communication, and tailored support to ensure successful integration of mental health services into primary care.

## Abstract (fr)

**Contexte:**

Le modèle de soins collaboratifs (Collaborative Care Model, CoCM) intègre les soins de santé mentale en soins primaires au sein d’équipes pluridisciplinaires associant médecins généralistes, infirmiers de coordination (care managers, CM) et professionnels de santé mentale consultants. Il constitue une approche éprouvée pour répondre à ces enjeux. En France, une première adaptation de ce modèle, le projet SESAME, est déployée en soins primaires depuis 2021.

**Méthodes:**

Des entretiens semi-directifs ont été menés auprès de l’ensemble des professionnels de santé impliqués dans SESAME afin d’examiner les facilitateurs et les obstacles rencontrés au cours de la première année de mise en œuvre. Les données ont été analysées selon une démarche qualitative combinant analyse thématique manuelle et analyse lexicométrique à l’aide du logiciel ALCESTE.

**Résultats:**

Les résultats ont mis en évidence quatre classes de discours. Les principaux facteurs facilitants concernaient une expérience préalable de collaboration, l’utilisation d’outils numériques de communication efficaces et une bonne compréhension des rôles. Les obstacles relevaient de difficultés techniques, d’ambiguïtés persistantes dans la définition des rôles et d’incompréhensions concernant les modalités d’inclusion et d’orientation dans le dispositif. Le caractère structuré du parcours d’inclusion et la rapidité d’accès aux soins ont été largement appréciés, mais des tensions persistaient autour des processus d’orientation et des responsabilités professionnelles. La prise en charge financière du dispositif ainsi que son caractère moins stigmatisant ont également été identifiés comme des bénéfices.

**Conclusion:**

Cette étude apporte des éclairages sur les dynamiques précoces de mise en œuvre d’un modèle de soins collaboratifs en santé mentale en soins primaires dans le contexte français. Elle souligne la nécessité de protocoles clairs, d’échanges réguliers entre professionnels et d’un accompagnement adapté pour soutenir l’intégration des soins de santé mentale en soins primaires.

## Introduction

Anxiety and depressive disorders are among the most prevalent and disabling mental health (MH) conditions globally [1,2].

In primary care settings, a significant proportion of consultations involve MH issues, with many cases remaining undiagnosed or masked by comorbid physical conditions [3,4,5,6]. General practitioners (GPs) are thus at the forefront of mental health care (MHC) delivery, despite limited time, resources, and training in MH [3,7,8]. They face multiple challenges in providing comprehensive MHC, including making accurate diagnoses, choosing appropriate treatment strategies, and ensuring adequate patient follow-up. They also report significant difficulties in establishing effective collaboration with MH professionals (MHP), particularly regarding access to psychiatric consultations and psychological interventions [9,10,5]. These challenges are compounded by systemic barriers to care, including stigma [11], cost for patients, and limited availability of specialized MH resources [12,13,14,15].

The Collaborative Care Model (CoCM) has emerged as a promising approach for integrating MH services into primary care settings. Implemented worldwide since the 90s and supported by over 90 randomized controlled trials [16,17], the model includes a team of professionals working together: primary care providers (typically GPs), behavioral health care managers (CMs), and mental health consultants. It features measurement-based treatment (using standardized questionnaires) through a structured management plan, systematic patient follow-up using a registry to track treatment progression, and regular systematic caseload reviews where MHP and CMs discuss patient cases and treatment plans. This approach ensures enhanced communication between healthcare professionals through shared documentation, team meetings, and ongoing supervision.

A gap between proven efficacy and context-specific implementation highlights a critical area for research [17,18]. Implementation research, which examines how interventions function in real-world settings, in comparison to efficacy studies that show what can work under ideal conditions, remain necessary in novel contexts - such as the French healthcare system which is characterized by structural and cultural specificities [19]. Until recently, limited financial coverage for care provided by private psychologists under the national health insurance system restricted access to MH services. However, France’s robust public insurance coverage generally distinguishes it from systems like those in the United States, where financial barriers to care are more pronounced [20]. The system also faces significant workforce challenges, including shortages of GPs and psychiatrists [21,22,23], and marked inequalities in their geographic distribution across the country [24,19]. France has notably high rates of psychotropic medication prescriptions, with GP responsible for approximately 90% of these prescriptions [25,26].

The SESAME (*Soins d’Equipe en SAnté MEntale*, i.e. Mental Health Team Care) project represents the first implementation of CoCM for common mental disorders (depressive and anxiety disorders) in French primary care settings. This team-based approach integrates GPs, nurses with at least five years of previous professional experience in psychiatry as care managers (noted CM in the text), and remotely supervising psychiatrists (PSY). SESAME’s deliberate choice to employ experienced nurses with a background in psychiatry as CMs reflects an intentional strategy to leverage specialized knowledge of the psychiatric sector within primary care settings. SESAME aims to provide integrated MHC for patients through rapid access to MH support, structured follow-up, and interprofessional collaboration. The program ensures full financial coverage for care provided by CMs, removing out-of-pocket costs for these consultations and enhancing accessibility for patients with limited financial resources.

## Implementation context

SESAME was launched in November 2021 by Versailles Hospital and Institut Montaigne across four primary care practices of varying size and organization in Yvelines, Île-de-France. In France, primary care operates primarily through independent fee-for-service practices with limited systematic collaboration between GPs and mental health specialists. Patients typically access psychiatrists or psychologists through direct referrals, with long waiting times and limited care coordination.

The project strategically chose the Yvelines for its diverse contexts (urban, rural, and semi-rural) and socially mixed population. The department has a lower GP density than the national average (292 vs. 339 per 100,000 inhabitants) and marked socioeconomic disparities, with poverty rates ranging from 7.2% to 27.8% across areas [27].

Program governance: The project was led by Versailles Hospital (implementation leadership) and Institut Montaigne (coordination, strategic decisions, governance advocacy). Clinical supervision was provided by psychiatrists, while practice-level coordination was facilitated by designated GP champions (GPs acting as SESAME reference persons within their practice setting) and CMs. The programme is supported by a mixed-methods, multidisciplinary evaluation, combining quantitative monitoring and qualitative implementation analysis conducted by an external academic team.

Implementation strategies: SESAME mobilised a set of core implementation strategies targeting both clinical practice and interprofessional collaboration. Before launch, GPs, CM and psychiatrists attended several online training modules covering CoCM principles, use of PHQ 9-GAD 7, SESAME’s digital monitoring tool, and follow up procedures. Patient identification relied on a brief PHQ 2 screening combined with the GP’s clinical judgement, followed by a standardised inclusion form completed by the GP and transmitted to the CM via secure messaging or GP software. Decision support tools specified eligibility thresholds for PHQ 9 and GAD 7 and clinical criteria, while weekly case review meetings between the CM and the psychiatrist were used to adjust treatment plans, particularly for new or complex cases; pharmacological treatment was adapted by GPs on the basis of psychiatric advice, with GPs remaining the formal prescribers.

Ongoing supervision and monitoring were ensured through weekly case reviews (3–4 hours) and site level meetings (monthly to quarterly) involving GPs, the CM and the psychiatrist. These activities relied on a dedicated SESAME patient monitoring software, implemented uniformly across sites, which was jointly used by CMs and psychiatrists to prepare case lists and regularly update symptom scores, follow-up visits and clinical status. In contrast, GPs continued to use their usual electronic health record (EHR) systems, which differed between practices and were not interoperable with the SESAME tool. Communication between team members used a combination of formal and informal channels. Beyond scheduled case reviews, CMs could contact the psychiatrist for ad hoc clinical advice, and additional training sessions were organised on request, particularly for GPs.

Outcomes evaluated during year 1 included implementation fidelity, professional experiences (current study) and satisfaction, patient experience and enrollment rates, and preliminary clinical outcomes (not reported here).

This study focuses on SESAME’s early implementation phase, offering insights into the processes and challenges encountered during the first year (0–12 months) of CoCM integration. While international research has extensively addressed implementation dynamics, the facilitators and barriers specific to this formative stage remain underexplored in the literature [28,29,30,31,32]. As part of a broader three-year longitudinal study [33], this analysis investigates the initial dynamics within the unique context of the French healthcare system. Responding to a gap identified by Proctor et al. (2011) [34] and Saldana (2014) [35], who noted that implementation research often emphasizes mature phases (>24 months), this study highlights the critical importance of understanding early-stage processes. By examining healthcare professionals’ experiences during SESAME’s first year, this work provides actionable insights for policymakers, healthcare providers, and researchers aiming to implementing CoCMs in diverse healthcare systems, while establishing a foundation for future analyses of SESAME’s longer-term evolution.

## Ethical considerations

This study was conducted in accordance with the Declaration of Helsinki and approved by a National Ethics Committee. Participants provided oral informed consent after being briefed on the study’s objectives and methods. They were informed of their right to withdraw at any time. Data were anonymized during transcription and analysis, with identifiable information stored securely. Participants received feedback on preliminary findings and will receive a written summary of the final results post-publication.

## Method

### Study design and approach

The qualitative study employed in-depth interviews to assess the implementation of SESAME. This approach is well-suited for examining complex implementation processes and capturing nuances of individual experiences. An inductive methodology was chosen to generate concepts and theories from field data, rather than testing predetermined hypotheses, which is particularly relevant for this exploratory study in a context where CoCM implementation is understudied.

This study did not include individuals with lived experience of MH problems or of using the SESAME programme. We acknowledge that this is a limitation, as the perspectives of those directly affected are critical to informing meaningful and contextually grounded research, and future work within the broader evaluation will seek to integrate service user perspectives more systematically.

### Participants and recruitment

SESAME was implemented across four pilot sites in the Yvelines area, selected by Versailles Hospital and Institut Montaigne to represent varying organizational structures, sizes, and practice environments. All four pilot sites were included in this qualitative evaluation:

– Site 1 - multidisciplinary group practice (MGP = collaborative healthcare settings where general practitioners work alongside other healthcare professionals): 7 GPs, 1 CM, 1 PSY
– Site 2 - MGP: 2 GPs, 1 CM, 1 PSY
– Site 3 - MGP - 3 GPs, 1 CM*, 1 PSY*
– Site 4 - three distinct solo GP practices 3 GPs (one per practice), 1 CM*, 1 PSY*
  *CM and psychiatrist shared between site 3 and site 4.

In MGP settings (sites 1–3), CMs were on-site intermittently while psychiatrists provided remote supervision. In solo practices (site 4), both the CM and psychiatrist worked entirely off-site (itinerant CM across practices, remote psychiatrist).

All the 21 healthcare professionals involved in the SESAME pilot (15 GPs, 3 psychiatrists, 3 CMs), were invited to participate. The SESAME project coordinator sent an initial email introducing the research to all professionals and provided their contact details to the researcher. The researcher then contacted each professional individually via email and phone to schedule interviews, emphasizing voluntary participation and confidentiality. All 21 professionals agreed to participate and were interviewed. One GP chose to withdraw from the SESAME experiment, their interview was therefore excluded from our analysis, resulting in a final sample of 20 participants.

### Data collection

Semi-structured interviews were conducted between April and September 2022, three to eight months post-implementation, lasting an average of 43 minutes (range: 25–73). Interviews were conducted in-person (15), by phone (4), or video conference (1), based on participant preference. Our interviews explored various aspects of the entry phase, including participants’ professional backgrounds, motivations for joining the program, understanding of the program’s framework, the use of information systems, and the overall quality of care provided. All interviews were conducted by the first author (IB).

### Data analysis

Interviews were recorded, fully transcribed verbatim (331 pages), and analyzed using a mixed approach combining manual thematic analysis and computer-assisted lexicometric analysis using ALCESTE (*Analyse des Lexèmes Co-occurrents dans les Énoncés Simples d’un Texte*) software [36,37].

Step 1 - preliminary manual thematic analysis: Two researchers independently reviewed the transcripts to identify major themes emerging from the data. Through iterative comparison and consensus discussion, seven (7) major themes were identified (Annex 1). This preliminary thematic framework served as the foundation for subsequent ALCESTE coding.

Step 2 - variable coding for ALCESTE: The corpus was prepared for lexicometric analysis by coding two types of variables: (a) thematic variables - the seven themes identified in Step 1 were coded throughout the transcripts to mark text segments corresponding to each theme; (b) participant characteristic variables - nine socio-professional variables were coded to examine their influence on discourse patterns (Table 1).

**Table 1:** Variables coded for analysis.


VARIABLE	CATEGORIES

Profession	GP, CM, Psychiatrist

Gender	Male, Female

Years of experience	<5 years, 5–15 years, >15 years

Experience in institution	Yes, No

Parallel activity	Yes, No

Type of practice structure	MGP, Solo practice

Number of SESAME sites*	1 site, 2 sites

GP champion**	Yes, No

Inclusion in SESAME	First wave (Oct 2021), Second wave (Feb 2022)


*Notes:* *Only applicable to CMs and psychiatrists. **Refers to a GP acting as SESAME program reference within their practice setting.

Step 3 - ALCESTE lexicometric analysis: the coded corpus was then analyzed with ALCESTE, which performed a descending hierarchical classification of text segments based on co-occurring vocabulary patterns, correspondence factor analysis to visualize relationships between classes, and co-occurrence calculations (Chi-square: χ²) to identify the most characteristic word forms for each class.

The ALCESTE analysis of the corpus, comprising 2,898 text segments, achieved a 76% classification rate (2,193 classified segments), indicating good stability. ALCESTE identified four lexical classes representing distinct discourse patterns within the corpus. For each class, only word forms with the highest χ² values were retained; based on threshold visualization, the top seven reduced forms per class were selected as the most representative (Annex 2).

The four classes were then interpreted by returning to the original transcripts to examine their thematic content and their relationship to participant variables (Table 1). This analytical approach enhanced rigor by combining inductive thematic interpretation with objective lexicometric pattern detection, ensuring comprehensive exploration of themes and minimizing the risk of overlooking significant elements [37,38,39].

## Results

### Sample characteristics

Table 2 presents the demographic and professional characteristics of the study participants.

**Table 2:** Characteristics of study participants (N = 20).


CHARACTERISTIC	n (%)

**Profession**	

General Practitioner	14 (70%)

Psychiatrist	3 (15%)

Care Manager	3 (15%)

**Gender**	

Female	14 (70%)

Male	6 (30%)

**Age range**	

25–34 years	5 (25%)

35–44 years	7 (35%)

45–54 years	5 (25%)

55–64 years	1 (5%)

65 years and older	2 (10%)

**Years of experience**	

0–5 years	1 (5%)

5–15 years	9 (45%)

More than 15 years	10 (50%)

**Type of practice**	

Multidisciplinary group practice (MGP)	14 (70%)

Solo practice	4 (20%)

Group and solo practice	2 (10%)

**Institutional experience**	

Yes	11 (55%)

No	9 (45%)

**Parallel activity**	

Yes	4 (20%)

No	16 (80%)

**Number of SESAME sites (NUR and PSY only, n = 6)**	

1 site	4 (67%)

2 sites	2 (33%)

Champion GP*** (GP only, n = 14)	

Yes	4 (28%)

No	10 (72%)

**Inclusion in SESAME**	

First wave (October 2021)	15 (75%)

Second wave (February 2022)	5 (25%)


*** Refers to a GP acting as SESAME program reference within their practice setting.

### Discourse classes

The ALCESTE analysis revealed four distinct discourse classes, presented here in their software-assigned numerical order, rather than by percentage weight. A detailed overview of the main topics, prominent words, and illustrative verbatims for each class, highlighting major key facilitators and barriers, is provided in Supplementary Table S1.

#### Class 1: Professional activity and experience (25.90% of classified segments)

This class focuses on participants’ professional backgrounds and collaborative experiences before SESAME implementation. Professionals described extensive experience, often in MGPs or at the hospital, highlighting a pre-existing interest in collaborative work. This prior collaborative culture was seen as a significant facilitator for SESAME implementation. The class also revealed professionals’ motivations for joining SESAME, including reducing professional isolation and opening their practice.

#### Class 2: Monitoring tools and team dynamics (6.66%)

This class highlights the technological and organizational facets of SESAME implementation, focusing on practical collaboration aspects. The deployment of effective digital communication tools and shared medical records emerged as a key facilitator, particularly for GPs who valued these tools for facilitating exchanges and achieving significant time savings. However, participants also noted that the lack of interoperability between the SESAME monitoring tool and GPs’ existing EHR systems generated duplicated documentation and additional workload, especially for CMs. CMs reported challenges in accessing information and using these tools effectively. Despite acknowledging their value, they also deplored the lack of direct interactions, exacerbated by inconsistent access to physicians’ professional software.

The rapid feedback systems were also identified as a significant advantage for GPs. Regular formal and informal meetings with CMs and psychiatrists, supported by professional software, were appreciated for their added value compared to traditional care models.

However, insufficient training for CMs was identified as a barrier, as they had to develop their own training methods and rely on peer support due to the lack of formal training on the tools they were expected to use. Disparities in work organization between different sites, especially with physicians in isolated practice, posed additional challenges to implementation.

#### Class 3: Roles understanding and perception of the program (60.83%)

This class, representing the largest portion of discourse, focuses on understanding the roles of each professional in SESAME and the general perception of the program. SESAME is perceived by GP’s as effectively addressing their needs by alleviating concerns about limited MH expertise, time constraints, referral difficulties and patient’s financial barriers to specialized psychological support. The program’s financial coverage for patients and facilitated access to nursing care are highly valued. The rapid and less stigmatizing nature of SESAME are highlighted as significant benefits, making it an optimal pathway for patients not yet ready to engage with MH professionals. A clear understanding of roles emerged as a crucial facilitator for successful implementation. CMs played a key role as coordinators, ensuring smooth communication and care transitions. Regular team meetings between professionals were valued for clarifying the framework. Additionally, the perception of SESAME as a high-quality care model, the presence of psychiatrists as expert backup providing enhanced clinical confidence for GPs, and GPs’ existing receptiveness to providing psychological support, despite recognized limitations, were also identified as facilitators.

However, challenges in defining role boundaries, particularly between CMs and psychologists, especially when they practice in the same location, were noted. Although psychologists were not part of the SESAME team, some GPs and patients struggled to distinguish between the work settings of a SESAME CM and a private psychologist. Furthermore, the need for precise inclusion and exclusion criteria was emphasized to avoid errors in patient referrals and ensure effective collaboration. The risk of GP disengagement due to unclear or misaligned referral processes was highlighted, as GPs sometimes delegated too quickly to CMs without fully engaging in the care process, often due to their time constraints and limited training.

#### Class 4: Patient inclusion and care (6.61%)

The fourth class concentrates on the clinical aspects of SESAME, including patient inclusion criteria and care processes. The structured inclusion process, facilitated by questionnaires, was identified as a key facilitator, helping to standardize the inclusion process across practitioners. The benefit of psychiatric expertise was also highlighted, as it provided reassurance to GPs.

However, challenges were noted in referral processes, particularly for specific disorders or treatments. Some GPs struggled to adapt to the new framework, finding it difficult to develop the reflex of identifying suitable patients for SESAME. Furthermore, there was a tendency to broaden the inclusion criteria beyond the program’s official focus, which led to difficulties for CMs in managing cases that did not strictly fit the SESAME framework.

## Discussion

This qualitative study provides critical insights into the first-year implementation of the SESAME CoCM for depression and anxiety in French primary care settings. Our results reveal several key facilitators and barriers consistent with international findings on CoCM implementation [30,40,41,42] in the following dimensions: pre-existing interest in collaborative and multifaceted work, monitoring tools and communication benefits and challenges, roles understanding and perception of SESAME as meeting clinical needs, and patient inclusion and care.

As our study’s focus on the entry phase (0–12 months) where most implementation research focuses on mature phases (>24 months) [34,35], it’s interesting to find the elements already described, with a few specifics linked to the set-up [30,40,41,42,43].

### Lessons Learned from Early Implementation

SESAME’s first year of implementation in France’s fragmented primary care system yielded several critical lessons for CoCM adaptation in similar contexts. A primary lesson concerns technology infrastructure: interoperability cannot be assumed in fee-for-service systems with heterogeneous information systems. The lack of system integration created double documentation burdens that threatened sustainability, while the absence of formal Information Technology (IT) training forced CMs to self-learn platforms. This reveals that technology readiness requires both technical integration and structured training protocols.

Organizational context emerged as a crucial moderator of implementation success. Solo practices faced greater coordination challenges than MGPs, highlighting that CoCM implementation strategies cannot be uniform across practice settings. Practices with lower organizational capacity may require additional external coordination support or phased implementation approaches.

Despite initial role definitions, confusion between CMs and psychologist functions persisted. This led to inappropriate referrals and patterns of GP over-delegation driven by time constraints and role misunderstandings rather than clinical assessment. This demonstrates that role boundaries require ongoing team discussion, structured case reviews, and iterative adjustment.

The strategic decision to employ CMs with a psychiatric background as care managers facilitated smoother collaboration with psychiatrists and GPs and helped reduce patient stigma around MH engagement. Financial coverage for patients addressed a critical access barrier for low-income populations. However, this benefit created an unintended pattern where GPs sometimes delegated patients based on inability to pay for private psychologists rather than on clinical complexity, potentially diluting the program’s focus on cases most appropriate for collaborative care.

Finally, SESAME demonstrated that collaborative culture requires structural activation, not just professional motivation. While pre-existing interest in teamwork facilitated initial adoption, sustainable collaboration required dedicated coordination time, structured case review meetings, formal training on the CoCM model, and iterative adjustments based on team feedback.

### Alignment with International CoCM Evidence

These implementation dynamics align with international CoCM research while revealing context-specific adaptations. The importance of organizational readiness through pre-existing collaborative culture mirrors US experiences where “organizational readiness” emerged as a critical precursor to successful implementation [41].

Role boundary challenges between CMs and psychologists represent a recurring theme in Spanish [40] and UK implementations [42], suggesting this is a universal CoCM implementation challenge.

The perception of SESAME as meeting GPs’ needs through rapid access to care and expert support echoes Spanish findings [40] on how CoCM improves depression management by reorganizing processes and providing timely specialist input. SESAME’s ability to provide structured pathways for patient care was particularly valued by GPs struggling with referral delays and limited access to psychiatrists.

By involving psychiatrists as consultants, SESAME ensures timely psychiatric input while maintaining GPs’ central role in patient management, offering a valuable lesson for countries facing similar workforce constraints and geographic disparities. Furthermore, SESAME’s implementation across diverse primary care settings (MGPs and private solo practices), highlights its adaptability to a fragmented healthcare landscape, though solo practices required additional coordination support to achieve comparable outcomes.

### Limitations and strengths

Our study has limitations that should be considered when interpreting the results. The specific organizational structures and funding mechanisms of the French healthcare system may limit direct comparisons with international experiences. For instance, unlike in the USA and UK, where CoCM has often been implemented within more integrated primary care systems (e.g., UK’s NHS talking therapies), the French primary care landscape remains largely fragmented, with independent fee-for-service practices, no pre-existing infrastructure for systematic GP–psychiatrist collaboration, and limited interoperability between information systems. Additionally, the absence of patient and health system administrator perspectives restricts the comprehensiveness of our findings. Future research should incorporate these viewpoints to gain deeper insights into implementation processes.

Key strengths of this study include the near-exhaustive inclusion of eligible professionals, the breadth of perspectives captured across sites, the timing of data collection close to the start of SESAME (a formative phase that is rarely examined in depth), and a rigorous qualitative analysis combining independent double coding, consensus-based interpretation and lexicometric methods.

## Conclusion

The first-year implementation of the SESAME CoCM in French primary care settings highlights both its potential and the challenges of adapting such models to a fragmented healthcare system. Key facilitators included the pre-existing interest in collaborative work among healthcare professionals, GPs’ perception of SESAME as addressing their needs, and the central role of CMs in fostering interprofessional collaboration. However, barriers such as role ambiguity, technological challenges, and maintaining GP engagement underscore the complexities of implementing CoCM in diverse organizational contexts.

Building directly on these findings and lessons learned, several actionable recommendations emerge:

– Invest in interoperable IT systems to facilitate communication across fragmented care settings and ensure equitable access to patient information for all team members.
– Facilitate regular team-based discussions to clarify referral criteria between CM and psychologists, based on clinical complexity, patient needs and available resources, recognising that CMs provide structured self-management support while psychologists are mobilised as specialised resources for patients with more complex psychological needs.
– Provide targeted training for CM in motivational interviewing, behavioral activation, and care coordination, addressing skill gaps identified during early implementation and supporting their role as the central liaison between GPs, psychiatrists, and other specialists.
– Create mechanisms to maintain GP engagement, including clear referral protocols, regular feedback loops, and ongoing communication to ensure balanced task-sharing and sustained involvement in CoCM processes.

By examining early-phase dynamics in a longitudinal framework, this study contributes valuable knowledge to CoCM implementation research. Future studies will build on these findings by exploring how facilitators and barriers evolve over time, informing strategies for scaling SESAME across France and other healthcare systems facing similar challenges.

## Additional Files

The additional files for this article can be found as follows:

10.5334/ijic.9845.s1 Supplementary Table S1. Facilitators and barriers to SESAME implementation: insights from ALCESTE analysis.

10.5334/ijic.9845.s2 Annexes. Annex 1 and 2.
