# Supplementary material for: Early Implementation of Collaborative Care in France: Facilitators and Barriers from the First Year of the SESAME Program
Source: Int J Integr Care. 2026 Oct 1;26(4):1. doi: 10.5334/ijic.9845 (PMC13635668; doi:10.5334/ijic.9845)
Supplement: Supplementary file 1 [file ijic-26-4-9845-s1.pdf]

Supplementary Table S1. Facilitators and barriers to SESAME implementation: insights from ALCESTE analysis

Class	Main topic	Most prominent words	Facilitators and barriers most illustrative verbatims
Class 1<br>25.90%	Professional activity and experience	#year, #private practice, #office, #set up, #action, #hospital, #service	**Facilitators:**<br>- **Prior collaborative experience and team-oriented professional culture**<br>*"In fact I've always been in a #private practice, i've never set up a practice on my own i've always worked in collaboration with other doctors but not with other healthcare professionals". (GP1)*<br>- **Multifaceted professional motivation**<br>*"You permanently work alongside specialists whom you consult for opinions, you see, when you enter an emergency #service, to find myself alone in an #office where I feel a bit isolated, it seemed interesting to me to be able to continue team work." (GP9)*<br>*"(in) the hospital, we are fragmented and that's really what I didn't want. [...] It's also about leaving the #hospital hierarchy where frankly I was fed up with the 'little bosses'. " (CMNUR3)*<br>*"When I thought 'I have to leave', I also thought I have to find things to do outside... we see people, we are useful to the public in #private practice but not in the public service network or things like that anymore... and yes, wanting to vary my activity a bit." (PSY2)*
Class 2<br>6.66%	Monitoring tools and team dynamics	#doctolib, #file/record, #message, #computer/IT, #tool, #software, #note	**Facilitators:**<br>**I. Technological and communication facilitators**<br>- ***Effective digital communication tools, monitoring record, and shared medical records***<br>*"They (the CM)She can record her consultation in Hello Doc, which is the professional #software common to all healthcare professionals in the MGP, and so during a next meeting with the patient, we see what the nurse has observed and what her problem is, if there is one, and what #message theyshe wants to pass on to us as a professional, so that's very interesting". (GP3)*<br>- ***Efficient communication and feedback systems for GPs***<br>*"I get feedback quite quickly, after the first or first two interviews, this feedback is systematically transcribed in the patient's #file, because that's important too, and it allows us not to have to make phone calls all the time." (GP6)*<br>*"I think the program is very well done because, as I said, we have formal and informal meetings with the nurse, we have professional #software support, and once a month or every two months, either in person or via Zoom... we can talk directly to the psychiatrist. So, that's really an invaluable comfort that we would never have with a traditional MGPCMP because we never get any feedback or patient information with an MGPCMP, in fact." (GP3)*<br>**II. Professional role and implementation framework facilitators**<br>- ***Support system created by care managersnurses***

			"Even the nurses, I know they somewhat trained themselves on the job, but with a lot of camaraderie among them, so they helped each other a lot. At the very beginning, A. and B. (nurses) took all the #tools, tried to appropriate them for themselves, and did a great job." (PSY1)<br><br>**Barriers :**<br><br>**I. Technological and communication barriers**<br><br>- ***Communication challenges between professionals = lack of direct exchanges***<br>*"Because once I'm at Y., we communicate about everything, but when I'm at X., I have trouble communicating with Y., and then at X., communication is done via #Doctolib****, but there are no direct exchanges." (CMNUR1)*<br><br>- ***Difficulties in accessing patient files***<br>*"At X., I don't have access to patient #files at all, so they arrive... I have raw patients, I have no history, no treatment... well, I just have my referral sheet and... and then I clear up the situation." (CMNUR1)*<br><br>- ***Collaborative and monitoring tools perceived as time-consuming and lacking ergonomics***<br>*"Honestly, from the beginning, this has been difficult for me because I feel this #tool isn't finished... it crashes, and since I'm very bad at computers, I don't know if it's the bug or me, and I waste a lot of time wondering if it's the #tool that's bugging or if it's me. So honestly, it's unbearable". (CMNUR3)*<br><br>- ***Absence of a unified information system***<br>*"if there was just a common #software, we would waste much less time, on transcribing everything, because they (GPs) would have access to the psychiatrist's notes, they would have access to all the elements, because we are forced to make transmissions on the #software, take another ticket for ... and so I think it would facilitate things." (CMNUR1)*<br><br>**II. Professional role and implementation framework barriers**<br><br>- ***Insufficient training for the care managersnurses***<br>*"Training is a real concern because, in fact, we didn't receive training with the #tools we're asked to use, and these are things we've never seen before. So, we did training in pairs with my colleague, with books, with tutorials, with... creating our own #tools. Honestly, training should be indispensable." (CMNUR3)*<br><br>***** Doctolib: commercial online scheduling and messaging platform widely used in French primary care.*
Class 3<br>60.83%	Roles understanding and perception of the program	#psychologist, #patient, #care, #see, #refer, #take, #need	**Facilitators (mostly for GPs) :**<br><br>**I. GP receptiveness to psychological support**<br><br>*"Overall, we do a lot of supportive therapy... well, we can't really call it therapy because I'm not a #psychologist, but it's more like supportive consultations". (GP15)*<br><br>**II. Professional role and implementation framework facilitator**<br><br>- ***Clear understanding of roles***<br>*"I think theyshe [the nurse] is a mediator - a tripartite one - between patients, GPs, and psychiatrists. TheyShe (CM) acts as a relay, transmitting*

			information and feeding back to both the GP, who referred the patient, and the psychiatrist to monitor the evolution of #care and treatments." (GP10)<br><br>*"It's an assistant (psychiatrist), an intervener who intervenes at the end of the chain, meaning... as GPs, we see the patient first. If we think we can #refer them to the nurse, then of course, we'll #lrefer them to the nurse first. They (CM)She proceeds step by step, conducts an interrogation, evaluates the patient's needs, and provides care, etc. They (CM)She feeds back information to us, and we implement a treatment. If the treatment doesn't evolve positively or if we need more precise and specific information about mental health, then I think theirher intervener is the psychiatrist." (GP3)*<br><br>- ***Care managersNurses as key coordinators***<br>*"They (CM)She has access to my schedule, and theyshe can schedule an appointment for the #patient or, in any case, ask the secretariat to do so, so that I can see them more quickly. In that case, theyshe will notify me in advance to say, 'Well, there's such and such a #patient that you'll see sooner because of this and that.' So, I would say that's where the nurse is... the master of time." (GP8)*<br><br>*"[...] It's going well because we have feedback since theyshe (CMthe nurse) presents it to B. (the psychiatrist) so theyshe gives us the feedback, the guidelines in fact to help us also in the #care so either request for psychological advice or... adaptation of the drug treatment, so it's also a help". (GP2)*<br><br>- ***Clear understanding of the framework***<br>*"It's true that we have #psychologists here too, who attended the first meetings on the SESAME program because it was important to show them that it wouldn't replace psychologists, so it was important to show them how it worked... and as a result, some patients are referred to psychologists, depending on the patients' income, but it evolves in that sense when there's a need." (GP1)*<br><br>*"this visit to people who may be reluctant at first sight to a treatment or other, and suddenly to say well wait we'll give ourselves time you'll discuss it, and then the nurse theyshe talks about it with a psychiatrist and we discuss it again together, I think it promotes a therapeutic alliance for the #patients". (GP13)*<br><br>- ***Recognition of GPs' limitations in providing mental health care by GP's (time constraints, expertise boundaries, referral difficulties)***<br><br>*"Well, I'm not a #psychologist, I'm not a psychiatrist, I'm not a psychotherapist, so I can try to support and help patients become aware, but I'm not a #psychologist... We already have many complex consultations with multiple reasons for consultation, and talking about mental health, well, it takes time." (GP12)*<br><br>*"It was the difficulty I have in #referring certain people who need follow-up with a psychiatrist, in fact. We have a big referral problem." (GP7)*<br><br>- ***Regular team meetings for framework clarification***<br>*"I spoke more with, A. the SESAME nurse, to see how, in fact, how the #patients arrived in front of her, whether they were rather... positive, let's say, in a... or if they were a bit... hesitant, how to present her, how theyshe presented themherself to the patients, to be... to be a bit in the same*

discourse, in fact, so that there is continuity, and to be in a logic also for the patient, so that he sees that yes, we work together, and that we agree on the functioning." (GP8)

"[...] these meetings are interesting because they allow us to learn from other patients who have been included, the difficulties that have arisen around other patients we don't know but which finally allow us to... imagine other #care management, other difficulties, other... and the solutions that have been brought to them." (GP9)

III. Clinical practice

- ***Rapid access to care***

"It responds precisely to someone who doesn't feel well and who #needs to, I would say, consult or be seen, to be taken care of quickly." (GP7)

- ***Less stigmatizing care through care managernurse intervention***

"It responds to the patient's reluctance, I mean, about going to see a nurse rather than a #psychologist, it's not the same image, in fact, for the patient, it doesn't have the same connotation, so there is more acceptability to seeing a nurse than a psychologist. Sometimes, there are patients where I think: in the long run, they will need that, but it can be a way to get there..." (GP8)

- ***Enhanced clinical confidence for GPs through shared responsibility and expert backup***

"It's true that the psychiatrist is also really in backup, and it's true that for the case reviews, it's useful for me to see what can be improved in terms of therapeutic management (#care), whether medication or psychotherapy. So, it's a plus because we have easy and quick help rather than referring to an external and often unknown psychiatrist for me." (GP4)

- ***Perception of SESAME as a high-quality care model***

"It's an incredible opportunity for the patient. Because the patient is much better taken #care of by the nurse and the psychiatrist than by the GP who, once again, doesn't have this latitude in terms of time". (GP3)

- ***Financial coverage for patients***

"She (the patient) needed a #psychologist support , and when I proposed this system to her, I immediately saw a small smile appear because she was happy to find someone who would say that the nurse would call her within 48/72 hours to schedule an appointment for the week. And, uh, she felt reassured right away, especially since my consultation was difficult for her to pay for. When I told her that everything would be covered, there was a third smile, and she really felt reassured and taken care of. So, there are cases where it's really very good, and that was perfect." (GP7)

"At the medical house, we have patients who generally have the means, so it's true that often the referral is rather direct to a #psychologist... it's true that often those who are referred to the SESAME network are those who don't have money (laughs) well, I... uh... because, in fact, those who need psychotherapy really need psychotherapy... so they are directly referred to #psychologists if they have the means and are willing and agreeable..." (GP15)

Barriers :

II. Professional role and implementation framework barriers

			- ***Role ambiguity and interprofessional boundary issues (car managersnurses vs psychologists)***<br>*"From the moment you follow a patient who's going to talk... you're also in a psychotherapeutic relationship, which is normal. It's not a criticism or anything, you see. So, what about... well, that's it. For me, today, it brings confusion. Because the patient will say it himself. And so, in the SESAME framework, it raises a question for me... we confuse the patient for him, it (SESAME nurse) also serves as a #psychologist, what they say, in fact. They say it, yes. So, what about the #psychologist...?" (GP10)*<br><br>*"what's the difference between seeing the nurse and seeing the #psychologist? Sometimes it's a bit blurred, you see. That is, patients who take the nurse for... for the #psychologist, I mean they have a good relationship with the nurse, so they're happy, they look forward to seeing the nurse again to discuss and so on, when I usually referred the patients to the #psychologist. So, what's the added value of the nurse compared to the #psychologist on this aspect, huh?" (GP10)*<br><br>- ***Referral framework misunderstanding / misusing***<br>*"Because at the beginning, I didn't understand the program and I referred #patients... in parallel to the psychologist of the MGP and to the nurse..." (GP3)*<br><br>*"[...] after that, I still have a bit of trouble... making the distinction between referring to the SESAME nurse or directly to the #psychologists because we have three #psychologists at the medical house and patients who generally have the means, so it's true that often the referral is rather direct to a psychologist... it's true that often those who are referred to the SESAME network are those who don't have money (laughs) well, I... uh... because, in fact, those who need psychotherapy really need psychotherapy... so they are directly referred to #psychologists if they have the means and are willing and agreeable..." (GP15)*<br><br>*"For example, today I had a referral for a patient, so I called the gentleman, asked him if someone had explained the project to him, my role at the health center, or if I should explain it to him. He replied, 'No, I wasn't there, it was my mother who was at the appointment.' However, in the referral, I had a completed questionnaire, I had everything. But it's happened several times that we discuss this, and no, in fact, as long as we don't see patients face-to-face, we can't #refer them..." (CMNUR2)*<br><br>*"It's true that there is an evolution, certainly, also in the... in the #care management with this program, I would say that we could be, have, so to speak, a certain laziness - I say it with quotes - of relying on the program, well even if it may not be eternal, but not doing the hierarchical work by ourselves regarding patient management, that is to say that we could be led to rely on a program and... well, to abandon, I would say... [...] And it's true that it was a way to make our work easier, but yes, in quotes, maybe to "get rid of a certain task"." (GP4)*<br><br>**III. Clinical practice barriers**<br><br>- ***Risk of GP disengagement due to unclear or misaligned referral processes***<br>*"But then he told me yes, it was up to me to also do the sick leaves for as long as necessary, and there I said to myself but there I'm not in my role, in fact. My role, so to speak, is also to #refer, saying to them that they need to see a psychiatrist. So after that, it's where I changed a bit, so my habit, and*

			I said to myself well now, I can refer to this system only people who don't need a psychiatrist, and who just need a psychologist, in fact, that's it, and so theyshe [CMthe nurse] plays the role of a psychologist." (GP7)
Class 4<br>6.61%	Patient inclusion and care	#depressive, #anxious, #anxio, #syndrome, #disturb, #symptoms, #moderate	**Facilitators :**<br>**III. Clinical practice facilitators**<br>- ***Benefit of psychiatric expertise***<br>*"There are also elderly people who were introduced because they had anxiety scores, but it turned out to be masked #depression with a delirious mask, and they (the GPs) were also struggling with that. So, there were more direct exchanges that reassured them, in fact, they felt secure (the GPs)." (PSY3)*<br>- ***Structured inclusion process with questionnaires***<br>*"Regarding the criteria, it's the GP who administers them, whether it's the anxiety or #depression scale... so it's done with a questionnaire, either self-assessment or hetero-assessment... anyway, the goal is to try to prevent and detect anxiety-depressive disorders in the general population as early as possible." (PSY3)*<br>**Barriers :**<br>**II. Professional role and implementation framework barriers**<br>- ***Practice adaptation challenges***<br>*"At the beginning, it's true that it wasn't always easy, well, to get into the mindset of thinking about it, since we had to acquire a new reflex. So, first, we had to put in our minds that a patient presenting with #depressive or #anxious syndromes could be included, and we had to think to ask the little questions, [...]." (GP4)*<br>- ***Referral framework misunderstanding / misusing***<br>*"There isn't always a sufficient evaluation, to me, at the beginning. We don't necessarily have to agree with the somewhat narrowed-down side of mild-to-moderate anxiety-depressive syndrome, they (GPs) say we could broaden. It's been super complicated to tell them no actually severe addiction disorders no it's not SESAME, but they think that yes it could fit in." (CMNUR3)*<br>*"Indeed, we have included patients who were too... pathological, in the sense that they had suicidal risks or were psychotic, so we really need to stay on the right indication, mild to moderate anxiety-#depressive disorders." (GP14)*
