# Supplementary material for: Early Implementation of Collaborative Care in France: Facilitators and Barriers from the First Year of the SESAME Program
Source: Int J Integr Care. 2026 Oct 1;26(4):1. doi: 10.5334/ijic.9845 (PMC13635668; doi:10.5334/ijic.9845)
Supplement: Supplementary file 2 [file ijic-26-4-9845-s2.pdf]

Annexes

Annex 1: Major themes emerging from manual thematic analysis of SESAME implementation

Theme	Description
1. Professional context and prior experience	Participants' professional backgrounds and collaborative experiences before SESAME implementation.
2. Motivation for joining SESAME	Reasons why healthcare professionals joined the SESAME program, including addressing gaps in mental health care.
3. Initial perceptions of the program and understanding of each professional role	Early perceptions of SESAME and comprehension of roles among healthcare professionals.
4. Support for implementation	Factors facilitating implementation such as training, IT tools, monitoring systems, and human assistance.
5. Implementation process: adjustments, workload, and overall appreciation	Challenges and adaptations during implementation, including workload management and overall satisfaction with the program.
6. Interprofessional collaboration dynamics and adaptation post-case review	Evolution of interprofessional collaboration and adjustments following case reviews.
7. Patient management, inclusion, perceptions of quality of care	Aspects related to patient inclusion, management, and perceptions of care quality within SESAME.

Annex 2. Dendrogram of corpus classes with main words ranked by Chi-square strength.

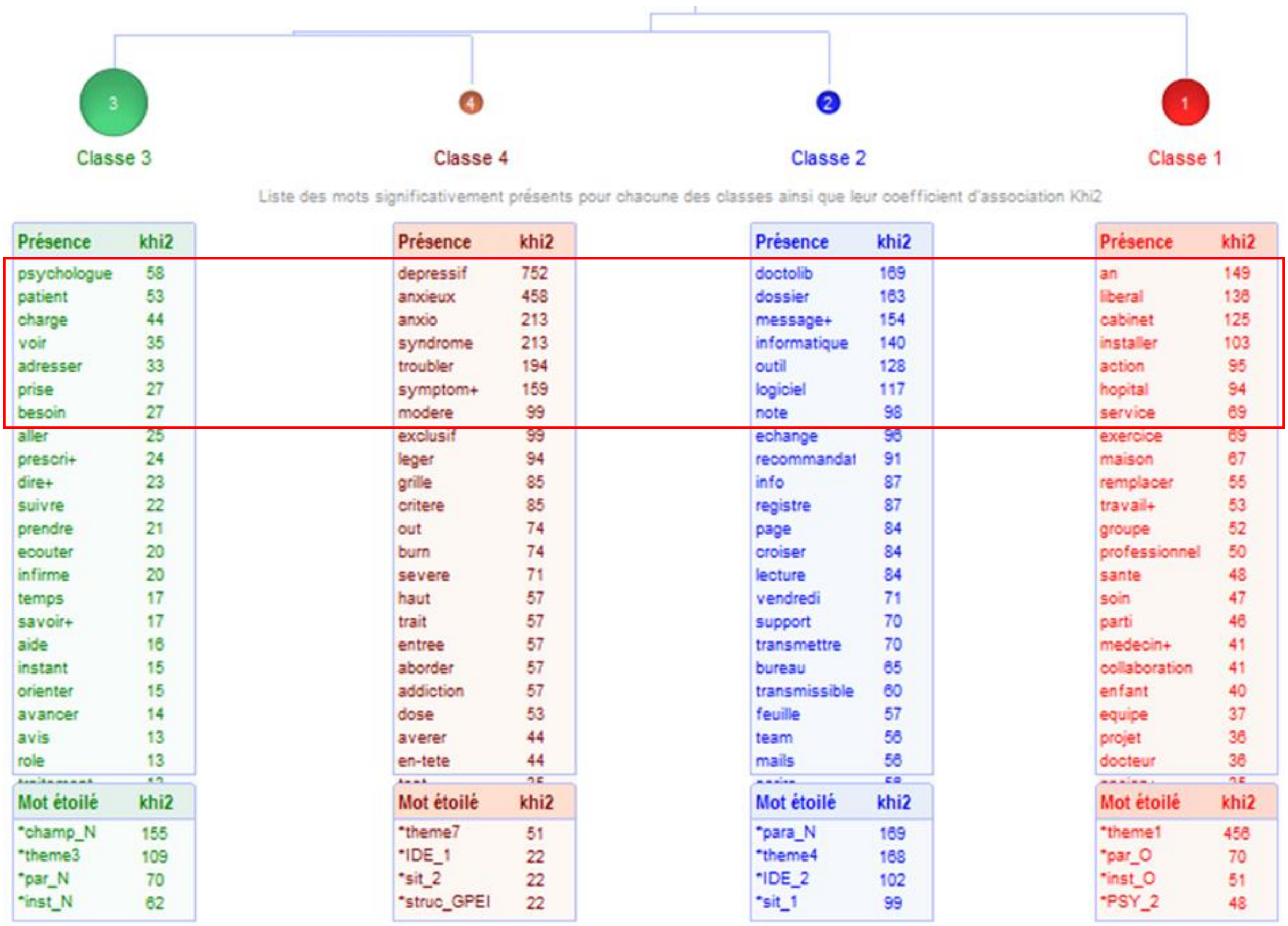

Présence (Classe 3)	khi2	Présence (Classe 4)	khi2	Présence (Classe 2)	khi2	Présence (Classe 1)	khi2
psychologue	58	depressif	752	doctolib	169	an	149
patient	53	anxieux	458	dossier	163	liberal	138
charge	44	anxio	213	message+	154	cabinet	125
voir	35	syndrome	213	informatique	140	installer	103
adresser	33	troubler	194	outil	128	action	95
prise	27	symptom+	159	logiciel	117	hopital	94
besoin	27	modere	99	note	98	service	69
aller	25	exclusif	99	echange	98	exercice	69
prescri+	24	leger	94	recommandat	91	maison	67
dire+	23	grille	85	info	87	remplacer	55
suivre	22	critere	85	registre	87	travail+	53
prendre	21	out	74	page	84	groupe	52
ecouter	20	burn	74	croiser	84	professionnel	50
infirme	20	severe	71	lecture	84	sante	48
temps	17	haut	57	vendredi	71	soin	47
savoir+	17	trait	57	support	70	parti	46
aide	16	entree	57	transmettre	70	medecin+	41
instant	15	aborder	57	bureau	65	collaboration	41
orienter	15	addiction	57	transmissible	60	enfant	40
avancer	14	dose	53	feuille	57	equipe	37
avis	13	averer	44	team	56	projet	36
role	13	en-tete	44	mails	56	docteur	36

Mot étoilé (Classe 3)	khi2	Mot étoilé (Classe 4)	khi2	Mot étoilé (Classe 2)	khi2	Mot étoilé (Classe 1)	khi2
*champ_N	155	*theme7	51	*para_N	169	*theme1	456
*theme3	109	*IDE_1	22	*theme4	168	*par_O	70
*par_N	70	*sit_2	22	*IDE_2	102	*inst_O	51
*inst_N	62	*struc_GPEI	22	*sit_1	99	*PSY_2	48

Legend:
The dendrogram illustrates four distinct discourse classes identified through ALCESTE analysis, with their respective sizes:
Class 1 (25.90%): Professional activity and practice
Class 2 (6.66%): Digital tools and communication
Class 3 (60.83%): Patient care and clinical practice
Class 4 (6.61%): Mental health conditions and symptoms

English translation of the most significant words by class, ranked by Chi-square value:

Class 1:	Class 2:	Class 3:	Class 4:
• *year (an)*<br>• *private practice (liberal)*<br>• *office (cabinet)*<br>• *set up (installer)*<br>• *action (action)*<br>• *hospital (hopital)*<br>• *service (service)*	• *doctolib (medical scheduling software)*<br>• *file/record (dossier)*<br>• *message (message)*<br>• *computer/IT (informatique)*<br>• *tool (outil)*<br>• *software (logiciel)*<br>• *note (note)*	• *psychologist (psychologue)*<br>• *patient (patient)*<br>• *care/management (charge)*<br>• *see (voir)*<br>• *refer (adresser)*<br>• *taking/handling (prise)*<br>• *need (besoin)*	• *depressive (dépressif)*<br>• *anxious (anxieux)*<br>• *anxiety (anxio)*<br>• *syndrome (syndrome)*<br>• *disorder (trouble)*<br>• *symptom (symptom)*<br>• *moderate (modéré)*

Note: The size of each class and Chi-square values indicate the relative importance and statistical significance of terms within their respective classes, reflecting key themes in healthcare professionals'.
